# Low thyroid function is associated with an increased risk of advanced fibrosis in patients with metabolic dysfunction-associated fatty liver disease

**DOI:** 10.1186/s12876-022-02612-3

**Published:** 2023-01-05

**Authors:** Hong Fan, Lili Li, Zhenqiu Liu, Pengyan Zhang, Sheng Wu, Xinyu Han, Xingdong Chen, Chen Suo, Liou Cao, Tiejun Zhang

**Affiliations:** 1 Department of Epidemiology, School of Public Health, Fudan University, Shanghai, 200032 China; 2 Key Laboratory of Public Health Safety (Fudan University), Ministry of Education, Shanghai, China; 3 Shanghai Institute of Infectious Disease and Biosecurity, School of Public Health, Fudan University, Shanghai, China; 4 Fudan University Taizhou Institute of Health Sciences, Taizhou, China; 5 Jiading District Central Hospital Affiliated Shanghai University of Medicine & Health Sciences, Shanghai, 200032 China; 6 State Key Laboratory of Genetic Engineering and Collaborative Innovation Center for Genetics and Development, School of Life Sciences, Fudan University, Shanghai, 200438 China; 7 Human Phenome Institute, Fudan University, 825 Zhangheng Road, Shanghai, China

**Keywords:** Fatty liver disease, MAFLD, Fibrosis, Low-normal thyroid function, Subclinical hypothyroidism

## Abstract

**Aims:**

Observational studies showed that low thyroid function may perturb liver function. We aimed to evaluate the association of low thyroid function with both metabolic dysfunction-associated fatty liver disease (MAFLD) and advanced hepatic fibrosis.

**Methods:**

Participants who underwent abdominal ultrasonography and thyroid function test in a Chinese hospital from 2015 to 2021were enrolled. Fibrosis-4 index (FIB-4) > 2.67 and/or non-alcoholic fatty liver disease fibrosis score (NFS) > 0.676 were used to define advanced fibrosis. Descriptive analyses were performed to characterize the epidemiology of MAFLD according to levels of thyroid-stimulating hormone (TSH). The logistic regression model was applied to estimate the association of low thyroid function with MAFLD and advanced fibrosis.

**Results:**

A total of 19,946 participants (52.78% males, mean age: 47.31 years, 27.55% MAFLD) were included, among which 14,789 were strict-normal thyroid function, 4,328 were low-normal thyroid function, 829 were subclinical hypothyroidism. TSH levels were significantly higher in MAFLD patients with a FIB-4 > 2.67 and /or NFS > 0.676 than their counterparts. The logistic regression model adjusted for age and sex showed that low-normal thyroid function increased the risk of MAFLD (odds ratio [OR] = 1.09; 95% confidence interval [CI] 1.01–1.18). Multivariable regression model adjusted for age, sex, body mass index, type 2 diabetes, and hypertension showed low-normal thyroid function increased the risk of advanced fibrosis in patients with MAFLD (FIB-4 > 2.67: OR = 1.41, 95% CI 1.02–1.93; NFS > 0.676: OR = 1.72, 95% CI 1.08–2.72).

**Conclusion:**

Elevated TSH concentrations are associated with advanced hepatic fibrosis, even in the euthyroid state.

## Introduction

Metabolic dysfunction-associated fatty liver disease (MAFLD) is the leading cause of chronic liver diseases worldwide, affecting approximately 30% of the global adult population [1–3]. MAFLD is a nomenclature that captures fatty liver disease (FLD) with metabolic dysfunctions. A previous study showed that the long-term complications of MAFLD have made it the most common cause of liver transplantation [4]. The etiology of MAFLD involves complex factors, such as obesity, hepatitis C infection, and diabetes [5]. Thyroid function has been proposed as one of the most important risk factors for its prominent effects on hepatic fatty acid and cholesterol synthesis [6, 7].

Thyroid hormones play a critical role in maintaining metabolic homeostasis throughout life and are intimately linked to the liver [6, 7]. Normal thyroid function is essential for maintaining liver metabolism; while thyroid disorders affect the clinical progression of liver disease [8]. In practice, thyroxine (T4), free T4 (FT4), and thyroid-stimulating hormone (TSH) can well reflect the decline of thyroid function, while TSH levels higher than the reference range usually suggest that the thyroid gland is underactive, as in low thyroid function, such as subclinical hypothyroidism (SCH) [9]. SCH is defined as elevated plasma TSH levels with normal FT4 levels, with a reported prevalence of 4–20% [10]. A previous study reported that of the patients visiting the clinic, those with both SCH and FLD were heavier and had more metabolic abnormalities than those with SCH and a normal liver [11].

For a long time, SCH has been defined as a TSH concentration of > 4.5 mIU/L with normal T4 and FT4 levels; however, current guidelines suggest that the TSH level cutoff value should be reduced to 2.5 mIU/L, which indicates low-normal thyroid function [12, 13]. By reducing the TSH level cutoff value to 2.5 mIU/L, more individuals in the general population will be classified with low thyroid function (including SCH and low-normal thyroid function). Unfortunately, recent studies investigating the prevalence of low-normal thyroid function among the general population and in patients with MAFLD, and the association between low thyroid function and both MAFLD and hepatic fibrosis, have been insufficient and have yielded inconsistent results [10, 14, 15]. Meanwhile, in China, the prevalence and other epidemiological characteristics of thyroid function and FLD are varied owing to changes in environmental, and socioeconomic factors, and the rising standard of living. Therefore, it is warranted to study the relationship between MAFLD and low thyroid function in the general population.

Here, we used data from Jiading District Central Hospital, Shanghai, China who underwent both abdominal ultrasonography and thyroid function examination to investigate the association of low thyroid function with both MAFLD and hepatic fibrosis. By using a relatively large population data set, this study provides a comprehensive analysis of low thyroid function, MAFLD, and hepatic fibrosis to address the current gap in the literature.

## Methods

### Populations and study design

This study included 21,308 adult individuals who underwent abdominal ultrasonography and thyroid function tests in a hospital (Jiading District Central Hospital Affiliated Shanghai University of Medicine &Health Sciences, Shanghai, China) from 2015 to 2021. Thyroid function was evaluated by laboratory determination of thyroxine (T4), free thyroxine (FT4), and thyroid stimulating hormone (TSH). In the recruitment of the study, individuals with a history of thyroid disease or treatment (e.g., thyroid nodules, thyroid cancer, hypothyroidism, and hyperthyroidism) were excluded according to the inpatient system, outpatient system, and self-report. Individuals with extreme values and missing aspartate aminotransferase (AST), alanine aminotransferase (ALT), albumin, and fasting glucose levels were excluded before the analysis. Participants who exceed the normal reference range of FT4 levels (FT4 < 9.01 pmol/L and FT4 > 19.04 pmol/L) and with TSH levels under 0.35 mIU/L were excluded. The study protocol was approved by the Human Research Ethical Committee of Shanghai University of Medicine &Health Sciences.

### Disease definition

Fatty liver disease (FLD) was diagnosed by clinicians based on the abdominal ultrasonography results. For patients with FLD, MAFLD was identified as the presence of FLD in addition to one of the following three criteria: 1. presence of type 2 diabetes mellitus (T2DM); 2. Individuals with body mass index (BMI) ≥ 24; 3. Individuals who had 2 metabolic risk abnormalities including arterial hypertension, hypertriglyceridemia, low high-density lipoprotein (HDL) cholesterol, and prediabetes [16]. Hypertension was defined as a systolic blood pressure of ≥ 130 mmHg or a diastolic blood pressure of ≥ 85 mmHg. Hypertriglyceridemia was defined plasma triglycerides ≥ 1.70 mmol/L. T2DM was defined as fasting glucose levels ≥ 7 mmol/L. Prediabetes was defined as fasting glucose levels ranging from 5.6 to 6.9 mmol/L. Individuals with plasma HDL-cholesterol < 1.0 mmol/L for men and < 1.3 mmol/L for women were considered with low high-density lipoprotein (HDL) cholesterol [16, 17]:

The probability of advanced fibrosis was estimated using the fibrosis-4 index (FIB-4) and non-alcoholic fatty liver disease (NAFLD) fibrosis score (NFS). The FIB-4 value of < 1.30 and/or NFS of < − 1.455 were used to exclude MAFLD patients with advanced fibrosis, and FIB-4 value of > 2.67 and/or NFS of > 0.676 were used to identify patients with advanced fibrosis of FLD [18]. The formula for quantifying NFS [19] and FIB-4 [20] is as follows: NFS = − 1.675 + 0.037 × age (years) + 0.094 × BMI (kg/m^2^) + 1.13 × impaired fasting glycemia or diabetes (yes 1, no 0) + 0.99 × AST/ALT − 0.013 × platelet (10^9^/L) − 0.66 × albumin (g/dL) and FIB-4 = (age [years] × AST [U/L])/ (platelet [10^9^/L] × (ALT [U/L])^1/2^).

Thyroid function parameters including TSH, FT4, and T4 levels were analyzed. Plasma thyroid hormone levels were measured in blood specimens collected by venipuncture during the physical examination. We used the normal reference ranges for thyroid function recommended by the manufacturers. Strict-normal thyroid function was defined as normal plasma TSH and FT4 levels (reference ranges: TSH 0.45 − 2.5 mIU/L and FT4 9.01–19.04 pmol/L) [21, 22]. Low thyroid function was defined by elevated TSH concentrations (> 2.5 mIU/L) but FT4 concentrations within the reference range [21, 22]. Among the patients with low thyroid function, low-normal thyroid function was defined as a TSH level of 2.5–4.5 mIU/L with a normal FT4 level and SCH was defined as a TSH level of > 4.5 mIU/L with a normal FT4 level.

### Statistical analysis

Baseline characteristics between groups were compared using the chi-square test, Student’s t-test, analysis of variance and Fisher’s exact test, as appropriate. Continuous variables are presented as mean ± standard deviation (SD) and categorical variables as numbers and percentages. Body mass index (BMI) was calculated as body weight (kg) divided by body height squared (m^2^). Overweight/obese was defined as individuals with a BMI of ≥ 24 kg/m^2^.

To assess whether low-normal thyroid function and SCH were associated with the risk of MAFLD and hepatic fibrosis, the individuals were categorized into three groups according to their TSH levels: TSH 0.35–2.5 mIU/L (strict-normal thyroid function), 2.5–4.5 mIU/L (low-normal thyroid function) and > 0.45 mIU/L (SCH) [12, 13, 22].

Multivariable Logistic regression models were used to identify the associations between low thyroid function and MAFLD, as well as two non-invasive fibrosis scores (FIB-4 and NFS) and advanced fibrosis. The covariates were set in three ways: model 1 was a univariable regression model; model 2 was adjusted for age and sex; model 3 was additional adjusted BMI, T2DM, and hypertension. Odds ratios (ORs) and 95% confidence intervals (CIs) were calculated using logistic regression models. Statistical significance was set at P < 0.05. All statistical analyses were performed using R statistical software version 4.0.3 (R Foundation for Statistical Computing, Vienna, Austria).

## Results

### Baseline characteristics of study populations among thyroid function groups

Finally, a total of 19,946 participants (52.78% males, mean age: 47.31 years, 27.55% MAFLD) were included. Among the 19,946 participants with a normal reference range of thyroid hormone, 14,789 (74.15%) had strict-normal thyroid function and 5,157 (25.85%) had low thyroid function, of which the latter was further subclassified into 4,328 with low-normal thyroid function (21.70%) and 829 with SCH (4.15%) (Table 1). Compared with patients with strict-normal thyroid function, those with low thyroid function were more likely to be older, females; with higher levels of total cholesterol, high-density lipoprotein (HDL), low-density lipoprotein (LDL), triglycerides, AST, fasting glucose; with lower levels of ALT, gamma-glutamyl transpeptidase (GGT), platelets count, albumin; and a higher prevalence of T2DM and hypertension. Compared with patients with low-normal thyroid function, those with SCH were more likely to be older and have higher AST, Alkaline phosphatase (ALP), fasting glucose, and lower platelet count and albumin. The prevalence of MAFLD showed no significant differences across the TSH grades (strict-normal, 27.43%; low-normal, 27.90%; and SCH, 27.99%) (Table 1).

**Table 1:** Characteristics of the study participants according to their TSH levels, n = 19,946

Variables	Strict-normal thyroid (n = 14,789)	Low thyroid function (n = 5,157)	*P *Value	Low thyroid function (n = 5,157)		
				Low-normal (n = 4,328)	SCH (n = 829)	*P *Value
Age, year	46.10 ± 15.69	50.78 ± 17.71	3.35e−62	49.84 ± 17.34	55.67 ± 18.75	3.23e−16
Male, %	56.17	43.03	2.49e−59	43.40	41.13	0.243
BMI, kg/m^2^	23.50 ± 3.32	23.50 ± 3.31	0.978	23.49 ± 3.34	23.49 ± 3.17	0.933
T2DM, %	5.74	7.24	1.26e−04	6.77	9.66	0.004
Hypertension, %	28.52	34.90	1.12e−17	34.18	38.67	0.015
Total cholesterol	4.81 ± 0.91	4.87 ± 0.97	8.88e−05	4.87 ± 0.96	4.92 ± 1.04	0.191
HDL cholesterol	1.32 ± 0.33	1.34 ± 0.34	5.97e−05	1.34 ± 0.34	1.33 ± 0.34	0.780
LDL cholesterol	2.97 ± 0.81	3.00 ± 0.85	0.040	2.99 ± 0.84	3.03 ± 0.91	0.287
Triglycerides	1.58 ± 1.23	1.67 ± 1.40	1.78e−04	1.66 ± 1.43	1.69 ± 1.22	0.545
ALT, U/L	23.67 ± 20.18	22.51 ± 20.25	4.02e−04	22.58 ± 20.75	22.16 ± 17.40	0.543
AST, U/L	20.97 ± 9.97	21.37 ± 10.14	0.0149	21.24 ± 10.25	22.03 ± 9.54	0.032
GGT, U/L	31.09 ± 31.39	29.32 ± 32.85	7.57e−04	29.25 ± 33.31	29.70 ± 30.39	0.701
ALP, U/L	67.81 ± 19.35	68.03 ± 22.30	0.523	67.48 ± 21.40	70.90 ± 26.36	4.50e−04
Glucose, mmol/L	5.27 ± 1.26	5.36 ± 1.31	1.26e−05	5.33 ± 1.27	5.52 ± 1.51	4.58e−04
Platelets 10^9^/L	218.81 ± 55.27	216.86 ± 59.63	0.039	218.00 ± 59.15	210.90 ± 61.78	0.002
Albumin, g/L	46.26 ± 2.57	46.07 ± 2.73	9.46e−06	46.12 ± 2.69	45.80 ± 2.92	0.003
MAFLD, %	27.43	27.90	0.520	27.89	27.99	0.988

*T2DM* Type 2 diabetes mellitus; *BMI* Body mass index; *HDL* High-density cholesterol; *LDL* Low-density cholesterol; *ALT* Alanine aminotransferase; *AST* Aspartate aminotransferase; *GGT* Gamma-glutamyl transpeptidase; *ALP* Alkaline phosphatase; *FLD* Fatty liver disease; Thyroid function: Strict-normal, TSH 0.35 − 2.5 mIU/L; low, TSH ≥ 2.5 mIU/L; low-normal, TSH 2.5–4.5 mIU/L; SCH, TSH > 4.5 mIU/L

### Baseline characteristics of MAFLD patients among thyroid function groups

Among the 5495 patients with MAFLD and normal reference range of thyroid hormone, 4056 (73.81%) had strict-normal thyroid function and 1439 (26.19%) had low thyroid function, of which the latter was further subclassified into 1207 (21.97%) with low-normal thyroid function and 232 (4.22%) with SCH (Table 2). Compared with patients with strict-normal thyroid function, those with low thyroid function were more likely to be older, females; with a higher prevalence of T2DM and hypertension; with higher levels of total cholesterol, HDL, fasting glucose, and lower levels of ALT, GGT, and albumin. Compared to patients with low-normal thyroid function, those with SCH were more likely to be older, with higher levels of fasting glucose and prevalence of T2DM, and lower levels of triglycerides. Compared to patients with strict-normal thyroid function, patients with low thyroid function with a higher proportion of advanced fibrosis (FIB-4 > 2.67: 6.48% vs. 3.39%; NFS > 0.676: 3.41% vs. 1.63%). Among patients with low thyroid function, FIB-4 values of > 1.30 (52.16% vs. 37.91%) and NFS values of > -1.455 (41.81% vs. 32.09%) were more prevalent in patients with SCH than in those with low-normal thyroid function (Table 2).

**Table 2:** Characteristics of the patients with MAFLD according to their TSH levels, n = 5495

	Strict-normal N = 4056	Low thyroid function N = 1439	*P *Value	Low thyroid function (TSH ≥ 2.5 mIU/L)		
				Low-normal N = 1207	SCH N = 232	*P *Value
Age	49.69 ± 15.46	54.84 ± 16.56	< 2.2e−16	54.02 ± 16.39	59.06 ± 16.82	3.48e−05
Male, %	75.62	57.09	< 2.2e−16	58.04	52.16	0.113
BMI	26.33 ± 2.81	26.33 ± 2.94	0.123	26.18 ± 2.97	26.18 ± 2.79	0.370
T2DM, %	12.52	15.08	0.016	14.17	19.83	0.035
Hypertension, %	46.91	52.97	9.36e−05	52.91	53.25	0.983
Total cholesterol	5.03 ± 0.95	5.07 ± 1.00	0.152	5.07 ± 0.99	5.07 ± 1.09	0.947
HDL cholesterol	1.12 ± 0.23	1.15 ± 0.26	2.35e−04	1.15 ± 0.26	1.16 ± 0.25	0.579
LDL cholesterol	3.15 ± 0.85	3.15 ± 0.89	0.912	3.14 ± 0.87	3.21 ± 0.97	0.273
Triglycerides	2.37 ± 1.67	2.46 ± 1.82	0.107	2.50 ± 1.92	2.24 ± 1.17	0.006
ALT, U/L	37.17 ± 27.42	34.13 ± 28.29	4.28e-04	34.40 ± 29.33	32.76 ± 22.07	0.329
AST, U/L	25.67 ± 13.06	25.55 ± 13.59	0.785	25.58 ± 14.08	25.43 ± 10.69	0.857
GGT, U/L	47.04 ± 40.94	43.30 ± 42.97	0.004	43.82 ± 44.62	40.60 ± 33.01	0.202
ALP, U/L	73.44 ± 19.17	72.73 ± 20.02	0.241	72.88 ± 19.72	71.93 ± 21.57	0.532
Glucose, mmol/L	5.75 ± 1.70	5.88 ± 1.75	0.019	5.83 ± 1.69	6.13 ± 2.01	0.032
Platelet, 10^9^/L	221.73 ± 55.35	221.22 ± 61.25	0.781	222.21 ± 61.91	216.05 ± 57.52	0.141
Albumin, g/L	46.63 ± 2.53	46.36 ± 2.65	0.001	46.38 ± 2.68	46.30 ± 2.50	0.675
FIB-4 > 1.30, %	29.58	40.21	1.87e−13	37.91	52.16	6.89e−05
FIB-4 > 2.67, %	3.39	6.48	7.54e−07	6.40	6.90	0.892
NFS > − 1.455, %	25.09	33.66	4.79e−10	32.09	41.81	0.005
NFS > 0.676, %	1.63	3.41	8.06e−05	3.49	3.02	0.868

T2DM, Type 2 diabetes mellitus; BMI, body mass index; HDL, high-density cholesterol; LDL, low-density cholesterol; ALT, alanine aminotransferase; AST, aspartate aminotransferase; GGT, gamma-glutamyl transpeptidase; ALP, alkaline phosphatase; FLD, fatty liver disease; Thyroid Function: Strict-normal, TSH 0.4 − 2.5 mIU/L; low thyroid function: TSH ≥ 2.5 mIU/L; low-normal, TSH 2.5–4.5 mIU/L; subclinical hypothyroidism, TSH > 4.5 FIB-4, fibrosis-4 index; NFS, non-alcoholic fatty hepatic fibrosis score

### The association between thyroid dysfunction and MAFLD

**Fig. 1:**
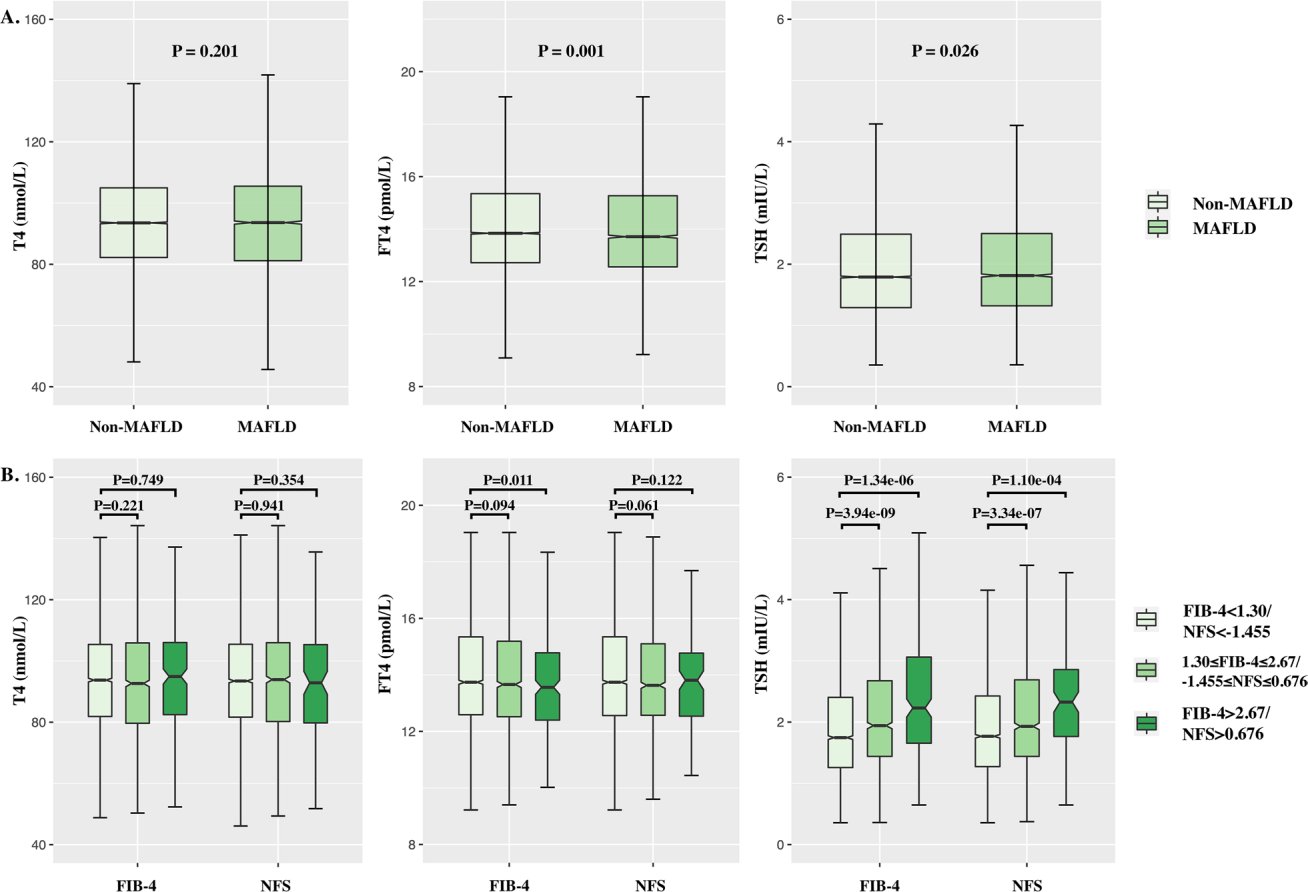
Boxplots of the associations between thyroid hormone levels (T4, FT4, TSH) and both MAFLD and hepatic fibrosis (using a non-invasive hepatic fibrosis scoring system). **A** 19,946 participants. **B** 5,495 patients with MAFLD. *FIB-4* Fibrosis-4 index; *FLD* Fatty liver disease; *NFS* Non-alcoholic fatty hepatic fibrosis score; *TSH* Thyroid-stimulating hormone; *T4* Thyroxine. *FT4* Free thyroxine

The plasma T4 showed no significant difference between non-MAFLD and MAFLD (T4, 94.03 nmol/L vs. 93.68 nmol/L;) (Fig. 1). The levels of FT4 and TSH showed a slight difference between non-MAFLD and MAFLD (FT4, 14.11 pmol/L vs. 14.01 pmol/L; TSH, 2.10 mIU/L vs. 2.17 mIU/L).

The univariable regression model did not detect any significant association between thyroid dysfunction and MAFLD risk. After adjusting for age and sex, the logistic regression analysis showed that low-normal thyroid function increased the risk of MAFLD (odds ratio [OR] = 1.09; 95% confidence interval [CI] 1.01–1.18) (Table 3). In the multivariable regression model additional adjusted for BMI, T2DM, and hypertension, the significant association between low-normal thyroid function and MAFLD disappeared.

**Table 3:** The associations between TSH levels and MAFLD, as well as the FIB-4 and NFS values in patients with FLD

		Low thyroid function		Low thyroid function			
				Low-nornal		SCH	
		OR (95% CI)	*P *Value	OR (95% CI)	*P *Value	OR (95% CI)	*P *Value
Non-MAFLD		Ref		Ref		Ref	
MAFLD	Model 1	1.02 (0.95, 1.10)	0.508	1.02 (0.95, 1.10)	0.549	1.03 (0.88, 1.20)	0.725
	Model 2	1.08 (1.00, 1.16)	0.043	1.09 (1.01, 1.18)	0.028	1.03 (0.87, 1.22)	0.702
	Model 3	1.01 (0.92, 1.11)	0.803	1.02 (0.93, 1.12)	0.690	0.99 (0.81, 1.20)	0.921
FIB-4 ≤ 1.30		Ref		Ref		Ref	
FIB-4 > 1.30	Model 1	1.60 (1.41, 1.81)	1.84e−13	1.45 (1.27, 1.66)	5.37e−08	2.59 (1.99, 3.39)	2.26e−12
	Model 2	1.00 (0.83, 1.19)	0.964	0.95 (0.79, 1.16)	0.632	1.23 (0.83, 1.83)	0.295
	Model 3	0.99 (0.83, 1.19)	0.943	0.95 (0.78, 1.15)	0.597	1.25 (0.85, 1.85)	0.266
FIB-4 > 2.67	Model 1	1.98 (1.50, 2.59)	7.85e−07	1.95 (1.46, 2.59)	4.97e−06	2.11 (1.19, 3.51)	0.006
	Model 2	1.31 (0.97, 1.77)	0.074	1.41 (1.02, 1.93)	0.033	0.92 (0.50, 1.63)	0.795
	Model 3	1.32 (0.97, 1.78)	0.072	1.41 (1.02, 1.93)	0.035	0.95 (0.51, 1.68)	0.874
NFS ≤ − 1.455		Ref		Ref		Ref	
NFS > − 1.455	Model 1	1.52 (1.33, 1.73)	4.44e−10	1.41 (1.23, 1.62)	1.54e−06	2.15 (1.63, 2.81)	3.13e−08
	Model 2	0.97 (0.81, 1.16)	0.745	0.97 (0.80, 1.18)	0.781	0.98 (0.67, 1.42)	0.904
	Model 3	0.92 (0.76, 1.13)	0.439	0.94 (0.76, 1.17)	0.595	0.86 (0.57, 1.28)	0.447
NFS > 0.676	Model 1	2.13 (1.46, 3.09)	7.47e−05	2.18 (1.46, 3.22)	9.76e−05	1.88 (0.78, 3.86)	0.119
	Model 2	1.41 (0.93, 2.10)	0.100	1.57 (1.02, 2.39)	0.036	0.79 (0.31, 1.75)	0.594
	Model 3	1.52 (0.97, 2.37)	0.065	1.72 (1.08, 2.72)	0.022	0.78 (0.29, 1.84)	0.592

*FIB-4* Fibrosis-4 index; *MAFLD* Metabolic dysfunction-associated fatty liver disease; *NFS* Non-alcoholic fatty hepatic fibrosis score, *TSH* Thyroid-stimulating hormone; *SCH* Subclinical hypothyroidism

Thyroid Function: Strict-normal, TSH 0.4–2.5 mIU/L; low thyroid function: TSH ≥ 2.5 mIU/L; low-normal, TSH 2.5–4.5 mIU/L; subclinical hypothyroidism, TSH > 4.5

Model 1 is a univariable regression model; model 2 is adjusted for age and sex; model 3 additionally adjusted for body mass index, type 2 diabetes, and hypertension

### The association between thyroid dysfunction and hepatic fibrosis in MAFLD patients

Compared with the low fibrosis score group (FIB-4 < 1.30 and/or NFS < − 1.455), the levels of T4 and FT4 showed no significant or slight difference with the medium (1.30 ≤ FIB-4 ≤ 2.67: and/or − 1.455 ≤ NFS ≤ 0.676) or high fibrosis score group (FIB-4 > 2.67 and/or NFS > 0.676). The levels of TSH were significant difference between FIB-4 < 1.30 and 1.30 ≤ FIB-4 ≤ 2.67 (2.0 mIU/L 3 vs. 2.44 mIU/L), FIB-4 < 1.30 and FIB-4 > 2.67 (2.03 mIU/L vs. 2.59 mIU/L), NFS < − 1.455 and − 1.455 ≤ NFS ≤ 0.676 (2.06 mIU/L vs. 2.44 mIU/L), and NFS < − 1.455 and NFS > 0.676 (2.06 mIU/L vs. 2.58 mIU/L) (Fig. 1).

The univariable regression model showed that SCH and low-normal thyroid function were associated with FIB-4 values of > 1.30 (low-normal: OR 1.45; 95%CI 1.27–1.66; SCH: OR 2.59; 95%CI 1.99–3.39) and NFS values of > − 1.455 (low-normal: OR 1.41; 95%CI 1.23–1.62; SCH: OR 2.15; 95%CI 1.63–2.81). These associations disappeared after adjusted covaries in the multivariable regression model. Low-normal thyroid function increased the risk of advanced fibrosis both in the univariable regression model and multivariable regression model. The multivariable regression model adjusted for age, sex, BMI, T2DM, and hypertension showed that low-normal thyroid function significantly increased the risk of advanced fibrosis in MAFLD patients (FIB-4 > 2.67: OR = 1.41; 95% CI 1.02–1.93; NFS > 0.676: OR = 1.72; 95% CI 1.08–2.72) (Table 3).

## Discussion

In the present study, we used real-world data to examine the association of TSH levels with MAFLD and hepatic fibrosis in patients with normal thyroid hormone levels. Our findings revealed that low-normal thyroid function was associated with an increased risk of advanced hepatic fibrosis in MAFLD patients, which has important implications for public health.

MAFLD is a nomenclature proposed in 2020 to replace the definition of NAFLD. Therefore, most of the current studies have focused on elucidating the associations between thyroid function and NAFLD. Generally speaking, NAFLD patients are always characterized by lower levels of T4 and FT4, and higher levels of TSH [23–30]. As a sensitive indicator of thyroid function, TSH levels have been studied extensively previously. Several epidemiological studies, conducted in various countries throughout the world, have shown an inverse association between TSH levels and the prevalence of NAFLD [6, 28, 31], and these associations have further been validated in longitudinal studies [32] and mendelian randomization studies [33, 34]. In clinical, elevated TSH levels indicates hypothyroidism or SCH. Hypothyroidism affects 0.6–12% of women and 1.3–4% of men worldwide and has been widely proven to be associated with NAFLD and its advanced fibrosis risk [6, 35, 36]. It is worth noting that, in addition to hypothyroidism, SCH, which had a higher prevalence, was also associated with the risk of NAFLD, non-alcoholic steatohepatitis, and advanced fibrosis [22, 26, 27, 37].

SCH is a common condition, with a reported prevalence of 4–20% [10]. In clinical practice, a TSH level of < 4.5 mIU/L has been applied to diagnose SCH for many years [12, 13]. Reducing the TSH cutoff level from 4.5 to 2.5 mIU/L to diagnose SCH means that more individuals in the general population will be classified with low thyroid function. In our study, low-normal thyroid function was much more prevalent than SCH (21.70% vs. 4.16% in the general population and 21.97% vs. 4.22% in patients with MAFLD). In addition, findings showed that low-normal thyroid function increased the risk of advanced fibrosis in two non-invasive fibrosis score systems, which suggests that elevated TSH within the normal range may be detrimental to health from a public health perspective.

Although many efforts have been made across the globe to elucidate the association between thyroid function and NAFLD, findings have yielded inconsistent results [38] and the evidence for MAFLD is insufficient. Our study based on a large sample size to supplement the research on thyroid dysfunction and MAFLD for current research. The diagnosis of FLD and the detection of other indicators were performed by professional physicians, which strengthens our study. However, this study had some limitations. First, FLD was diagnosed by ultrasound, and the advanced fibrosis was evaluated by noninvasive fibrosis score, which has low PPV and the established associations may actually be driven by something else than fibrosis. Second, selection bias may exist for the patients who underwent thyroid testing. Third, the study did not evaluate C-reactive protein, waist circumference, and insulin resistance, therefore, the prevalence of MAFLD is underestimated. Finally, the study population was from one hospital and cannot represent the general population. Therefore, Findings need to be validated using more sophisticated techniques (e.g., MRE and liver biopsies), and a more representative population.

In conclusion, our findings provide clues that elevated TSH within the normal range may also increase the risk of advanced fibrosis in patients with MAFLD, which has implications for clinical practice and public health.

## Data Availability

The datasets used and analyzed during the current study available from the corresponding author on reasonable request.

## References

[1] Le MH, Yeo YH, Li X, Li J, Zou B, Wu Y, Ye Q, Huang DQ, Zhao C, Zhang J (2021). 2019 global NAFLD prevalence: a systematic review and meta-analysis. Clin Gastroenterol Hepatol.

[2] Younossi ZM (2017). Long-term outcomes of nonalcoholic fatty liver disease: from nonalcoholic steatohepatitis to nonalcoholic steatofibrosis. Clin Gastroenterol Hepatol Offi Clin Pract J Am Gastroenterol Assoc.

[3] Estes C, Anstee QM, Arias-Loste MT, Bantel H, Bellentani S, Caballeria J, Colombo M, Craxi A, Crespo J, Day CP (2018). Modeling NAFLD disease burden in China, France, Germany, Italy, Japan, Spain, United Kingdom, and United States for the period 2016–2030. J Hepatol.

[4] Pais R, Barritt ASt, Calmus Y, Scatton O, Runge T, Lebray P, Poynard T, Ratziu V, Conti F (2016). NAFLD and liver transplantation: current burden and expected challenges. J Hepatol.

[5] Younossi Z, Anstee QM, Marietti M, Hardy T, Henry L, Eslam M, George J, Bugianesi E (2018). Global burden of NAFLD and NASH: trends, predictions, risk factors and prevention. Nat Rev Gastroenterol Hepatol.

[6] Sinha RA, Singh BK, Yen PM (2018). Direct effects of thyroid hormones on hepatic lipid metabolism. Nat Rev Endocrinol.

[7] Ritter MJ, Amano I, Hollenberg AN (2020). Thyroid hormone signaling and the liver. Hepatology.

[8] Jones DD, May KE, Geraci SA (2010). Subclinical thyroid disease. Am J Med.

[9] Zhou W, Brumpton B, Kabil O, Gudmundsson J, Thorleifsson G, Weinstock J, Zawistowski M, Nielsen JB, Chaker L, Medici M (2020). GWAS of thyroid stimulating hormone highlights pleiotropic effects and inverse association with thyroid cancer. Nat Commun.

[10] Cooper DS, Biondi B (2012). Subclinical thyroid disease. Lancet.

[11] Posadas-Romero C, Jorge-Galarza E, Posadas-Sánchez R, Acuña-Valerio J, Juárez-Rojas JG, Kimura-Hayama E, Medina-Urrutia A, Cardoso-Saldaña GC (2014). Fatty liver largely explains associations of subclinical hypothyroidism with insulin resistance, metabolic syndrome, and subclinical coronary atherosclerosis. Eur J Endocrinol.

[12] Brabant G, Beck-Peccoz P, Jarzab B, Laurberg P, Orgiazzi J, Szabolcs I, Weetman AP, Wiersinga WM (2006). Is there a need to redefine the upper normal limit of TSH?. Eur J Endocrinol.

[13] Wartofsky L, Dickey RA (2005). The evidence for a narrower thyrotropin reference range is compelling. J Clin Endocrinol Metab.

[14] van Tienhoven-Wind LJ, Dullaart RP (2015). Low-normal thyroid function and the pathogenesis of common cardio-metabolic disorders. Eur J Clin Invest.

[15] Mitchell F (2012). Thyroid function: low T_4_ levels a risk factor for fatty liver?. Nat Rev Endocrinol.

[16] Eslam M, Newsome PN, Sarin SK, Anstee QM, Targher G, Romero-Gomez M, Zelber-Sagi S, Wai-Sun Wong V, Dufour JF, Schattenberg JM (2020). A new definition for metabolic dysfunction-associated fatty liver disease: an international expert consensus statement. J Hepatol.

[17] Chen X, Chen S, Pang J, Tang Y, Ling W (2021). Are the different MAFLD subtypes based on the inclusion criteria correlated with all-cause mortality?. J Hepatol.

[18] Berzigotti A, Boursier J, Castera L, Cazzagon N, Friedrich-Rust M, Petta S, Thiele M, Tsochatzis E (2021). Easl clinical practice guidelines (Cpgs) on non-invasive tests for evaluation of liver disease severity and prognosis- 2020 update. J Hepatol.

[19] Angulo P, Hui JM, Marchesini G, Bugianesi E, George J, Farrell GC, Enders F, Saksena S, Burt AD, Bida JP (2007). The NAFLD fibrosis score: a noninvasive system that identifies liver fibrosis in patients with NAFLD. Hepatology.

[20] Kim D, Kim WR, Kim HJ, Therneau TM (2013). Association between noninvasive fibrosis markers and mortality among adults with nonalcoholic fatty liver disease in the United States. Hepatology.

[21] Jain RB (2017). Associations between the levels of thyroid hormones and lipid/lipoprotein levels: data from national health and nutrition examination survey 2007–2012. Environ Toxicol Pharmacol.

[22] Kim D, Yoo ER, Li AA, Fernandes CT, Tighe SP, Cholankeril G, Hameed B, Ahmed A (2019). Low-normal thyroid function is associated with advanced fibrosis among adults in the United States. Clin Gastroenterol Hepatol.

[23] van den Berg EH, van Tienhoven-Wind LJ, Amini M, Schreuder TC, Faber KN, Blokzijl H, Dullaart RP (2017). Higher free triiodothyronine is associated with non-alcoholic fatty liver disease in euthyroid subjects: the lifelines cohort study. Metabolism.

[24] Gökmen FY, Ahbab S, Ataoğlu HE, Türker B, Çetin F, Türker F, Mamaç RY, Yenigün M (2016). FT3/FT4 ratio predicts non-alcoholic fatty liver disease independent of metabolic parameters in patients with euthyroidism and hypothyroidism. Clinics (Sao Paulo).

[25] Tan Y, Tang X, Mu P, Yang Y, Li M, Nie Y, Li H, Zhu Y, Chen Y (2021). High-normal serum thyrotropin levels increased the risk of non-alcoholic fatty liver disease in euthyroid subjects with type 2 diabetes. Diabetes Metab Syndr Obes.

[26] Mantovani A, Nascimbeni F, Lonardo A, Zoppini G, Bonora E, Mantzoros CS, Targher G (2018). Association between primary hypothyroidism and nonalcoholic fatty liver disease: a systematic review and meta-analysis. Thyroid.

[27] Kim D, Kim W, Joo SK, Bae JM, Kim JH, Ahmed A (2018). Subclinical hypothyroidism and low-normal thyroid function are associated with nonalcoholic steatohepatitis and fibrosis. Clin Gastroenterol Hepatol.

[28] Ludwig U, Holzner D, Denzer C, Greinert A, Haenle MM, Oeztuerk S, Koenig W, Boehm BO, Mason RA, Kratzer W (2015). Subclinical and clinical hypothyroidism and non-alcoholic fatty liver disease: a cross-sectional study of a random population sample aged 18 to 65 years. BMC Endocr Disord.

[29] Xu C, Xu L, Yu C, Miao M, Li Y (2011). Association between thyroid function and nonalcoholic fatty liver disease in euthyroid elderly Chinese. Clin Endocrinol (Oxf).

[30] Guo W, Qin P, Li XN, Wu J, Lu J, Zhu WF, Diao QQ, Xu NZ, Zhang Q (2021). Free triiodothyronine is associated with hepatic steatosis and liver stiffness in euthyroid chinese adults with non-alcoholic fatty liver disease. Front Endocrinol (Lausanne).

[31] Eshraghian A, Hamidian Jahromi A (2014). Non-alcoholic fatty liver disease and thyroid dysfunction: a systematic review. World J Gastroenterol.

[32] Chung GE, Kim D, Kwak MS, Yim JY, Ahmed A, Kim JS (2021). Longitudinal change in thyroid-stimulating hormone and risk of nonalcoholic fatty liver disease. Clin Gastroenterol Hepatol.

[33] Qiu S, Cao P, Guo Y, Lu H, Hu Y (2021). Exploring the causality between hypothyroidism and non-alcoholic fatty liver: a mendelian randomization study. Front Cell Dev Biol.

[34] Fan H, Liu Z, Zhang X, Wu S, Shi T, Zhang P, Xu Y, Chen X, Zhang T (2022). Thyroid stimulating hormone levels are associated with genetically predicted nonalcoholic fatty liver disease. J Clin Endocrinol Metab.

[35] Piantanida E, Ippolito S, Gallo D, Masiello E, Premoli P, Cusini C, Rosetti S, Sabatino J, Segato S, Trimarchi F (2020). The interplay between thyroid and liver: implications for clinical practice. J Endocrinol Invest.

[36] Hollowell JG, Staehling NW, Flanders WD, Hannon WH, Gunter EW, Spencer CA, Braverman LE (2002). Serum TSH, T(4), and thyroid antibodies in the United States population (1988 to 1994): national health and nutrition examination survey (NHANES III). J Clin Endocrinol Metab.

[37] Chung GE, Kim D, Kim W, Yim JY, Park MJ, Kim YJ, Yoon JH, Lee HS (2012). Non-alcoholic fatty liver disease across the spectrum of hypothyroidism. J Hepatol.

[38] Jaruvongvanich V, Sanguankeo A, Upala S (2017). Nonalcoholic fatty liver disease is not associated with thyroid hormone levels and hypothyroidism: a systematic review and meta-analysis. Eur Thyroid J.

